# Trends in managing femoral neck fractures with arthroplasty in the ≥ 50 years age group

**DOI:** 10.12669/pjms.40.6.9135

**Published:** 2024-07

**Authors:** Syed Imran Bukhari, Noman Ali, Saleem Ullah, Mian Asad Ullah

**Affiliations:** 1 Dr. Syed Imran Bukhari, FCPS. Department of Trauma & Orthopedics, Lady Reading Hospital, Peshawar, Pakistan; 2 Dr. Noman Ali, FCPS. Department of Trauma & Orthopedics, Lady Reading Hospital, Peshawar, Pakistan; 3 Dr. Saleem Ullah, MBBS. Department of Trauma & Orthopedics, Lady Reading Hospital, Peshawar, Pakistan; 4 Dr. Mian Asad Ullah, MBBS. Department of Trauma & Orthopedics, Lady Reading Hospital, Peshawar, Pakistan

**Keywords:** Unipolar hemiarthroplasty, Bipolar hemiarthroplasty, Total hip arthroplasty

## Abstract

**Objective:**

To know about the trends in the management of neck of femur fractures with arthroplasty in patients ≥ 50 years.

**Methods:**

It is a retrospective cross-sectional study with data collection from Hospital Management Information System from 1^st^ January 2020 to 31^st^ July 2023. SPSS version 25 was used for data analysis. Mean & standard deviation was reported for quantitative variable & frequency and proportion were reported for qualitative variables. The cross- tabulations were performed to evaluate the association between the variables.

**Results:**

Total number of patients in this study was 305. Mean age was 67.80 ± 10.5 SD. Male to female ratio was 150:155. Co-morbidities were found in 126 patients. The surgical options used were Austin Moore prosthesis (64), Cemented Bipolar (36), Hybrid Total Hip Replacement (7), Non-cemented Total Hip Replacement (86), Cemented Total Hip Replacement (32), Uncemented Bipolar (71). Garden Type-2 fracture was noted in 33 patients, Type-3 in 170 patients and Type-4 in 87 patients. Cemented stem was used in 74 patients while 222 patients had non-cemented stem.

**Conclusion:**

One quarter of the patients had cemented stem implanted compared to three quarter of the patients who had non-cemented stem.

## INTRODUCTION

As life expectancy increases with advancing age due to improvements in personalized healthcare, the numbers of patients sustaining trivial trauma resulting in fracture are expected to increase.^1^ Gullberg et al. in 1997 calculated that the rate of femoral fractures will double from 1990 to 2025 worldwide and double again by 2050 with a range between 7.3 and 21.3 million fractures worldwide.^2^ Another analysis suggested an increase from 1.26 million in 1990 to 4.5 million by 2050.^3^

Geriatric patients are susceptible to hip fracture due to age-related changes in bone mineral density as well as other co-morbidities which impair bone quality. Hip fractures decrease patient independence, increase reliance on family and care-givers, put strain on national economy and have been associated with increased risk of mortality.^4^ As the population continues to age, the absolute number of falls and subsequently the number of fractures increase proportionately. There are a number of treatment options for fractures of the femoral neck. Typically, these fractures are classified as either non-displaced or displaced. Non-displaced fractures can be treated with internal fixation, although several studies have demonstrated internal fixation as a less optimal treatment, particularly in the elderly population.^5^,^6^ In dependent patients with limited life expectancy, surgeons tend to proceed with a hemiarthroplasty (HA), rather than total hip arthroplasty (THA). The HEALTH trial concluded that “the incidence of secondary hip procedure at 24 months was not different between HA and THA and THA provided clinically insignificant improvement over HA in function and quality of life over 24 months period^7^.”

Epidemiologic studies provide a reference for policymakers in healthcare delivery system to plan and mitigate the effects of injuries to reduce strain not only on national health but on national economy as well. There is scant epidemiological literature available nationally on this topic. Ahmad T et al. in their study in 2015 concluded that total hip replacement has a rising trend and hemiarthroplasties have a decreasing trend in the management of neck of femur fractures.^8^ We have tried to answer the following questions:

- How many elderly patients present with displaced neck of femur fractures?
- What implant options have been utilized?

## METHODS

**Fig 1:**
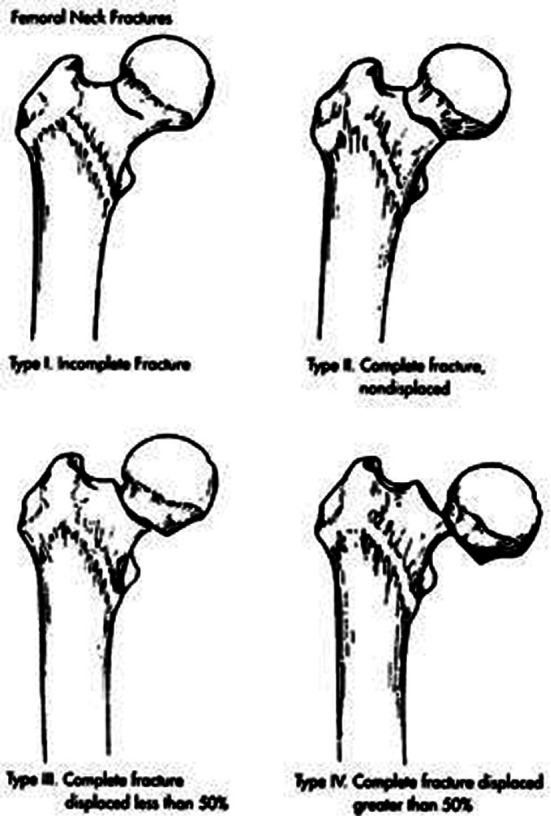
Garden classification.

This retrospective epidemiological study included all elderly patients (> 50 years) with a neck of femur fracture who were operated between January 2020 and July 2023 at the orthopedic and trauma surgery department of Lady Reading Hospital, Peshawar, Pakistan. The patient’s medical records were retrospectively reviewed using electronic medical records in the Hospital information system (HIS). All patients with documented neck of femur fracture (Garden Type-2,3,4), Fig.1, who were admitted and operated on with arthroplasty during the study period, were included. Garden Type-1 fracture was excluded. Multiple arthroplasty options were utilized, including AMP (Austin Moore Prosthesis), Bipolar hemiarthroplasty (cemented/non-cemented), Total hip arthroplasty (cemented/non-cemented).

### Ethical Approval

The Institutional Review Board of Lady Reading Hospital approved the research (REF. 921/LRH/MTI. Date: September 13, 2023.This study was carried out following the Helsinki Declaration.

The data were analyzed using SPSS version 25. The frequencies and proportions were presented as point estimates for categorical variables, while the mean ± SD was employed wherever necessary for quantitative variables. The cross- tabulations were performed to evaluate the association between the variables. Patients with missing data were included in the study but the missing data was excluded from calculations.

## RESULTS

**Fig 2:**
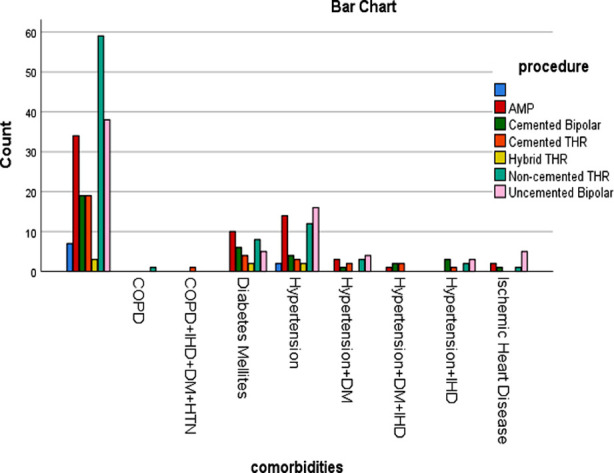
Co-morbidities vs procedure.

**Fig.3:**
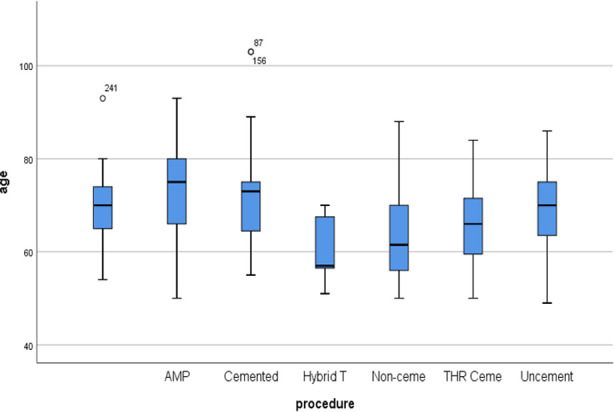
Age vs Procedure.

**Fig.4:**
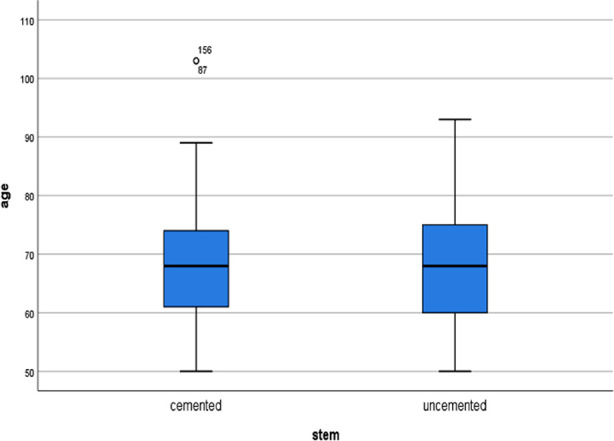
Age vs Stem.

Total number of patients in this study was 305. Mean age was 67.80 ± 10.51 SD. Male to female ratio was 150:155. One or multiple co-morbidities were found in 126 patients (Fig-2). The surgical options used were AMP (64), Cemented Bipolar (36), Hybrid THR (7), Non-cemented THR (86), Cemented THR (32), Uncemented Bipolar (71); Table-I. The time to surgery after injury was 12.38 ± 52.083 days. Garden Type-2 fracture was noted in 33 patients, Type-3 in 170 patients and Type-4 in 87 patients; different procedures were used in these fractures (Table-II). Dorr Type-A femur was observed in 9 patients (mean age 68.44 years), Type-B in 150 patients (mean age 67.86 years) and Type-C in 134 patients (mean age 67.66 years). Mean age of the patients receiving Austin Moore prosthesis (AMP) was 72.58 (69.68-75.47 CI) years, 71.61 (67.92-75.30 CI) years in those receiving cemented Bipolar, 60.86 (53.95-67.77 CI) years in Hybrid Total Hip replacement group (THR), 62.67 (60.84-64.51 CI) years in Non-cemented Total Hip replacement group, 66.16 (62.76-69.55 CI) years in cemented Total Hip replacement group, and 68.87 (66.73-71.01 CI) years in non-cemented Bipolar group (Fig.3). Of the available data, 74 patients had cemented stem while 222 patients had non-cemented stem. Mean age of the patients in non-cemented group was 67.54±10.48, while in cemented group, it was 68.28±10.50 (Fig-4). About 56% of patients in cemented group were female, while it was 50.22% in non-cemented group.

**Table-I:** Procedures.

Frequency		Percent
Missing	9	3.0
AMP	64	21.0
Cemented Bipolar	36	11.8
Hybrid THR	7	2.3
Non-cemented THR	86	28.2
THR Cemented	32	10.5
Uncemented Bipolar	71	23.3

Total	305	100.0

**Table-II:** Classification * procedure Crosstabulation.

Procedure									Total

			AMP	Cemented Bipolar	Hybrid THR	Non-cemented THR	THR Cemented	Uncemented Bipolar	
Classification		9	2	0	1	2	1	0	15
	2	0	4	0	4	7	0	18	33
	3	0	35	16	2	60	15	42	170
	4	0	23	20	0	17	16	11	87

Total		9	64	36	7	86	32	71	305

## DISCUSSION

Our results suggest that in patients ≥ 50 years old, non-cemented arthroplasty procedures are occurring more frequently than cemented arthroplasty. Hemiarthroplasty was carried out in 171 patients, while 125 patients had total hip arthroplasty. Out of 171 hemiarthroplasty procedures, 135 patients had non-cemented procedure, while 36 patients received cemented stems. Out of 125 total hip arthroplasty procedures, 86 patients had non-cemented stem, while 39 patients had cemented stem.

Fernandez et al. in their multi-center trial suggested that among patients 60 years of age or older with an intracapsular hip fracture, cemented hemiarthroplasty resulted in a modest but significantly better quality of life and a lower risk of periprosthetic fracture than non-cemented hemiarthroplasty.^9^ Lin FF et al. in their meta-analysis concluded that the available evidence indicates that compared with non-cemented hemiarthroplasty, cemented hemiarthroplasty achieved better postoperative hip function, less peri-operative fractures in displaced femoral neck fracture.^10^ There was no difference between the two groups with Harris Hip Score at one year, mortality, and complications. Migliorini F et al in their network meta-analysis concluded that total hip arthroplasty led to the highest Harris Hip scores and lowest rate of revision surgery compared to bipolar hemiarthroplasty and unipolar hemiarthroplasty. However, Bipolar hemiarthroplasty had the lowest dislocation rate when compared with Unipolar and total hip replacement. No significant differences in functional outcomes and complication rates were found between cemented and non-cemented implants.However, a tendency for lower mortality, revision and dislocation rates in cemented implants was evidenced.^11^ Parker MJ et al. concluded that their results support the use of a cemented hemiarthroplasty for the routine management of elderly patients with a displaced neck of femur fracture of the hip.^12^ The recent literature cited above on cemented vs non-cemented arthroplasty in elderly has favored cemented stems, contrary to our findings.

Fernandez et al reported that 69% of the patients were female in cemented group, while 66.8% patients were female in non-cemented group, compared to 56% female in cemented group and 50.22% in non-cemented group in the present study.^9^ In the study by Migliorini et al., 71% were women compared to 50.8% in the present study.^11^

The mean age in the study by Fernandez et al was 84.5 and 84.3 in cemented and non-cemented group respectively, compared to mean age of the patients in non-cemented group (67.54±10.48) and cemented group (68.28±10.50) in the present study.^9^ The meta-analysis by Lin FF et al included seven studies.^10^ Out of seven studies, six studies had reported mean age of more than 80 years, compared to 67.80 in the present study. The mean age of the patients in the study by Migliorini et al. was 77.2 years compared to 67.80 years in the present study.^11^ Mean age of the patients in the study by Parker MJ et al. was 85 years compared to 67.80 in this study.^12^ The mean age of the patients in the present study is relatively younger chronologically but younger age doesn’t explain the use of non-cemented stem as shown in Fig.4 in our present study, which shows that non-cemented stems have frequently been used in relatively elderly population as well. Mean age of the patients in the present study, receiving AMP was 72.58 years, 71.61 year in those receiving cemented Bipolar, 60.86 year in Hybrid THR group, 62.67 year in Non-cemented THR group, 66.16 year in cemented THR group, and 68.87 years in non-cemented Bipolar group. Our results show a mixed and inconsistent pattern. Of note is the finding that more elderly patients received non-cemented implant (AMP & non-cemented bipolar), while the guidelines recommend use of cement in elderly patients^13^,^14^. Our study shows that 9/9 Dorr A femurs received non-cemented stem, 130/150 Dorr B femurs received non-cemented stems while 81/134 Dorr C femurs received non-cemented stems. The focus of the present study is to report on the trends of managing displaced neck of femur fractures in our population. Subsequent studies need to be done on the outcomes of the procedure.

In the study by Ahmad T et al., hemiarthroplasty was the commonest procedure in 68% of patients.^8^ Our results show that 57.77% patients had hemiarthroplasty compared to 42.22% patients who had total hip arthroplasty. Jan-Erik Gjertsen argued in the editorial based on the results of HEALTH trial that we should probably be restrictive in the selection criteria for total hip arthroplasty for patients with hip fractures.^15^ Factors such as patient activity level, biologic age, and remaining life expectancy influence the choice of hemiarthroplasty vs total hip arthroplasty. HEALTH trial results were based on two years follow up, however long-term studies, preferably registry-based studies, are needed to address and inform on the best choice of implant in elderly population.

### Limitations

This is a retrospective data collection on the use of implant options in the management of femoral neck fractures in ≥50 years age group. Outcomes have not been assessed which can inform decision making in use of best implant.

## CONCLUSION

One quarter of the patients had cemented stem implanted compared to three quarter of the patients who had non-cemented stem.

### Authors` contribution:

**SIB** conceived and designed the study and is responsible for integrity of research. **SA and MAU** did data collection. **NA** did literature review.
